# 11,11-Diphenyl-11*H*-indeno­[1,2-*b*]quinoxaline

**DOI:** 10.1107/S2414314620012134

**Published:** 2020-09-08

**Authors:** Lin Chen, Jin Hu, Li-Li Wu, Hong-Shun Sun

**Affiliations:** a School of Biology and Environment, Nanjing Polytechnic Institute, Nanjing 210048, People’s Republic of China; b Targeted MRI Contrast Agents Laboratory of Jiangsu Province, Nanjing Polytechnic Institute, Nanjing 210048, People’s Republic of China; University of Aberdeen, Scotland

**Keywords:** crystal structure, indene, quinoxaline, indeno­[1,2-*b*]quinoxaline

## Abstract

In the title compound, C_27_H_18_N_2_, the mean planes of the pendant benzene rings are approximately perpendicular to one another, making a dihedral angle of 79.3 (5)°; the indeno­[1,2-*b*]quinoxaline ring system (r.m.s. deviation = 0.1197 Å) is twisted with respect to the pendant benzene rings by 70.0 (4) and 67.6 (3)°. Weak aromatic π–π stacking [centroid–centroid separation = 3.628 (2) Å] and C—H⋯π inter­actions occur in the crystal.

## Abstract 2

In the title compound, the mean planes of the pendant benzene rings are approximately perpendicular to one another, making a dihedral angle of 79.3 (5)°; the indeno­[1,2-*b*]quinoxaline ring system is twisted with respect to the pendant benzene rings by 70.0 (4) and 67.6 (3)°.

## Graphical abstract

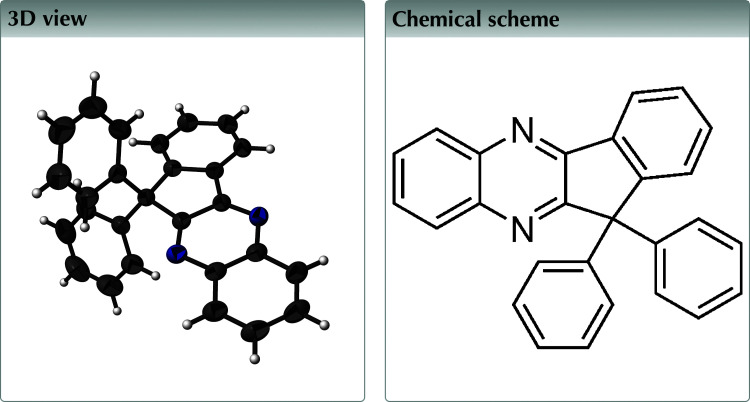

## Structure description

Some quinoxaline-based *N*-heteroacenes show a narrow band-gap, high thermal stability and aligned film morphology, which can be applied as the hole transport layers in quantum dot light-emitting diodes (QLEDs) (Bai *et al.*, 2015 ▸). As part of our work in this area, we now report the synthesis and crystal structure of the title indeno­[1,2-*b*]quinoxaline derivative.

**Figure 1:**
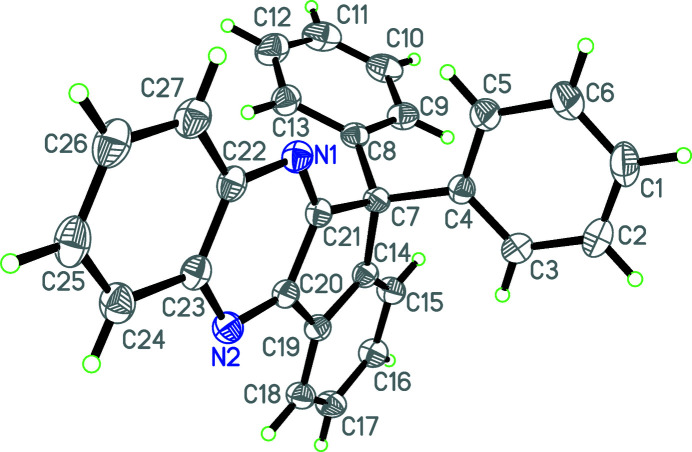
The mol­ecular structure of the title mol­ecule with displacement ellipsoids drawn at the 30% probability level.

The mol­ecular structure of the title compound is shown in Fig. 1 ▸. The pendant C1–C6 and C8–C13 benzene rings are nearly perpendicular to one another [dihedral angle = 79.3 (5)°] while the indeno­[1,2-*b*]quinoxaline fused ring system (N1–N2/C7,C14–C27) is twisted with respect to the C1–C6 and C8–C13 benzene rings, subtending dihedral angles of 70.0 (4) and 67.6 (3)°, respectively.

**Figure 2:**
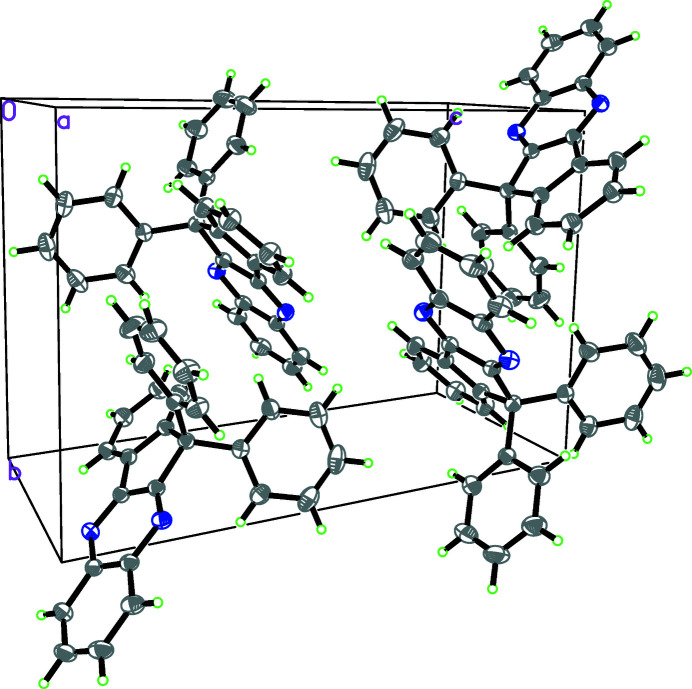
A packing diagram of the title compound.

In the crystal, weak *Cg*6⋯*Cg*6^i^ aromatic π–π stacking inter­actions [centroid–centroid separation = 3.628 (2), slippage = 1.717 Å, symmetry code: (i) = 1 − *x*, 1 − *y*, 1 − *z*; where *Cg*6 is the centroid of the C22–C27 benzene ring] link the mol­ecules into inversion dimers and weak C—H⋯π inter­actions link the dimers (Table 1 ▸, Fig. 2 ▸).

## Synthesis and crystallization

The title compound was prepared in three steps. In the first step, a mixture of 1*H*-indene-1,2,3-trione (3.20 g, 20 mmol) and benzene-1,2-di­amine (2.16 g, 20 mmol) in ethanol (100 ml) was heated to reflux under stirring for 5 h. 11*H*-Indeno­[1,2-*b*]quinoxalin-11-one (**1**) was obtained as yellow powder by filtering after cooling. Then, a solution of compound **1** (2.32 g, 10 mmol) in THF (30 ml) was added dropwise into a solution of phenyl­magnesium bromide (2.17 g, 12 mmol) in THF (30 ml). The mixture was heated to reflux with stirring for 12 h. This reaction was quenched by a saturated solution of NH_4_Cl and the inter­mediate 11-phenyl-11*H*-indeno­[1,2-*b*]quinoxalin-11-ol (**2**) was obtained by flash chromatography. In the last step, tri­fluoro­acetic acid (7 ml) was added dropwise to a solution of compound **2** (0.58 g, 2.50 mmol) in benzene (3 ml) and the mixture was heated at 50°C for 12 h. Then, the reaction mixture was transferred to an ice bath and NaOH was used to increase the pH of the solution to 10. After the reaction, DCM was used to extract the product and Na_2_SO_4_ was used as desiccant. The crude product was purified by flash chromatography to obtain a yellow powder product of the title compound. The total yield was about 15%. Single crystals of the title compound suitable for X-ray data collection were obtained by the slow evaporation of a methanol solution.

## Refinement

Crystal data, data collection and structure refinement details are summarized in Table 2 ▸.

## Supplementary Material

Crystal structure: contains datablock(s) I. DOI: 10.1107/S2414314620012134/hb4357sup1.cif

Structure factors: contains datablock(s) I. DOI: 10.1107/S2414314620012134/hb4357Isup2.hkl

Supporting information file. DOI: 10.1107/S2414314620012134/hb4357Isup3.cml

CCDC reference: 2026803

Additional supporting information:  crystallographic information; 3D view; checkCIF report

## Figures and Tables

**Table 1:** Hydrogen-bond geometry (Å, °) *Cg*2 and *Cg*3 are the centroids of the N1/N2/C20–C23 and C1–C6 rings, respectively.

*D*—H⋯*A*	*D*—H	H⋯*A*	*D*⋯*A*	*D*—H⋯*A*
C15—H15⋯*Cg*2^i^	0.93	2.92	3.728 (2)	145
C25—H25⋯*Cg*3^ii^	0.93	2.96	3.830 (3)	156

Symmetry codes: (i) 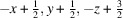 ; (ii) 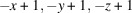 .

**Table 2:** Experimental details

Crystal data	
Chemical formula	C_27_H_18_N_2_
*M*_r_	370.43
Crystal system, space group	Monoclinic, *P*2_1_/*n*
Temperature (K)	296
*a*, *b*, *c* (Å)	9.642 (5), 11.407 (5), 17.858 (8)
β (°)	103.606 (5)
*V* (Å^3^)	1909.0 (16)
*Z*	4
Radiation type	Mo *K*α
μ (mm^−1^)	0.08
Crystal size (mm)	0.20 × 0.20 × 0.10

Data collection	
Diffractometer	Bruker APEXII CCD
Absorption correction	Multi-scan (*SADABS*; Bruker, 2004 ▸)
*T*_min_, *T*_max_	0.859, 1.000
No. of measured, independent and observed [*I* > 2σ(*I*)] reflections	11621, 4429, 3277
*R*_int_	0.023
(sin θ/λ)_max_ (Å^−1^)	0.653

Refinement	
*R*[*F* ^2^ > 2σ(*F* ^2^)], *wR*(*F* ^2^), *S*	0.043, 0.108, 1.03
No. of reflections	4429
No. of parameters	262
H-atom treatment	H-atom parameters constrained
Δρ_max_, Δρ_min_ (e Å^−3^)	0.15, −0.20

Computer programs: *APEX2* (Bruker, 2007 ▸), *SAINT* (Bruker, 2004 ▸), *SHELXT* (Sheldrick, 2015*a* ▸), *SHELXL* (Sheldrick, 2015*b* ▸) and *OLEX2* (Dolomanov *et al.*, 2009 ▸).

## full crystallographic data

### Crystal data

**Table:**

C_27_H_18_N_2_	*F*(000) = 776
*M**_r_* = 370.43	*D*_x_ = 1.289 Mg m^−^^3^
Monoclinic, *P*2_1_/*n*	Mo *K*α radiation, λ = 0.71073 Å
*a* = 9.642 (5) Å	Cell parameters from 3937 reflections
*b* = 11.407 (5) Å	θ = 2.4–27.4°
*c* = 17.858 (8) Å	µ = 0.08 mm^−^^1^
β = 103.606 (5)°	*T* = 296 K
*V* = 1909.0 (16) Å^3^	Block, colourless
*Z* = 4	0.20 × 0.20 × 0.10 mm

### Data collection

**Table:**

Bruker APEXII CCD diffractometer	3277 reflections with *I* > 2σ(*I*)
Graphite monochromator	*R*_int_ = 0.023
ω scans	θ_max_ = 27.6°, θ_min_ = 2.4°
Absorption correction: multi-scan (SADABS; Bruker, 2004)	*h* = −12→12
*T*_min_ = 0.859, *T*_max_ = 1.000	*k* = −14→14
11621 measured reflections	*l* = −23→18
4429 independent reflections	

### Refinement

**Table:**

Refinement on *F*^2^	Primary atom site location: dual
Least-squares matrix: full	Hydrogen site location: inferred from neighbouring sites
*R*[*F*^2^ > 2σ(*F*^2^)] = 0.043	H-atom parameters constrained
*wR*(*F*^2^) = 0.108	*w* = 1/[σ^2^(*F*_o_^2^) + (0.0447*P*)^2^ + 0.3316*P*] where *P* = (*F*_o_^2^ + 2*F*_c_^2^)/3
*S* = 1.03	(Δ/σ)_max_ = 0.001
4429 reflections	Δρ_max_ = 0.15 e Å^−^^3^
262 parameters	Δρ_min_ = −0.20 e Å^−^^3^
0 restraints	

### Special details

**Table:**

Geometry. All esds (except the esd in the dihedral angle between two l.s. planes) are estimated using the full covariance matrix. The cell esds are taken into account individually in the estimation of esds in distances, angles and torsion angles; correlations between esds in cell parameters are only used when they are defined by crystal symmetry. An approximate (isotropic) treatment of cell esds is used for estimating esds involving l.s. planes.
Refinement. H atoms were placed geometrically (C—H = 0.93 Å) and refined as riding atoms with *U*_iso_(H) = 1.2*U*_eq_(C).

### Fractional atomic coordinates and isotropic or equivalent isotropic displacement parameters (Å^2^)

**Table:**

	*x*	*y*	*z*	*U*_iso_*/*U*_eq_	
N1	0.51226 (11)	0.55738 (9)	0.65772 (6)	0.0394 (3)	
N2	0.23762 (11)	0.47436 (9)	0.57474 (6)	0.0395 (3)	
C1	0.5462 (2)	1.01277 (15)	0.63861 (11)	0.0695 (5)	
H1	0.5840	1.0786	0.6200	0.083*	
C2	0.4016 (2)	0.99914 (15)	0.62609 (11)	0.0716 (5)	
H2	0.3410	1.0565	0.5995	0.086*	
C3	0.34490 (17)	0.90057 (13)	0.65275 (9)	0.0541 (4)	
H3	0.2464	0.8923	0.6439	0.065*	
C4	0.43262 (13)	0.81433 (11)	0.69233 (7)	0.0375 (3)	
C5	0.57958 (15)	0.83006 (12)	0.70533 (9)	0.0495 (3)	
H5	0.6409	0.7734	0.7322	0.059*	
C6	0.63502 (18)	0.92863 (14)	0.67879 (11)	0.0631 (4)	
H6	0.7334	0.9382	0.6882	0.076*	
C7	0.37522 (12)	0.70131 (11)	0.72101 (7)	0.0350 (3)	
C8	0.44723 (13)	0.67853 (11)	0.80551 (7)	0.0382 (3)	
C9	0.45826 (15)	0.76998 (14)	0.85801 (8)	0.0495 (3)	
H9	0.4275	0.8445	0.8405	0.059*	
C10	0.51424 (17)	0.75184 (17)	0.93576 (9)	0.0620 (4)	
H10	0.5191	0.8135	0.9704	0.074*	
C11	0.56268 (18)	0.64278 (19)	0.96174 (10)	0.0707 (5)	
H11	0.6005	0.6304	1.0140	0.085*	
C12	0.55541 (18)	0.55199 (17)	0.91067 (10)	0.0667 (5)	
H12	0.5904	0.4786	0.9284	0.080*	
C13	0.49617 (15)	0.56892 (13)	0.83284 (9)	0.0504 (4)	
H13	0.4893	0.5063	0.7988	0.060*	
C14	0.21179 (13)	0.70151 (11)	0.70965 (7)	0.0363 (3)	
C15	0.12920 (14)	0.76476 (12)	0.74945 (8)	0.0450 (3)	
H15	0.1717	0.8172	0.7879	0.054*	
C16	−0.01721 (15)	0.74878 (13)	0.73120 (9)	0.0486 (3)	
H16	−0.0727	0.7907	0.7580	0.058*	
C17	−0.08276 (14)	0.67165 (13)	0.67389 (9)	0.0486 (3)	
H17	−0.1814	0.6630	0.6622	0.058*	
C18	−0.00211 (14)	0.60742 (12)	0.63394 (8)	0.0434 (3)	
H18	−0.0454	0.5555	0.5953	0.052*	
C19	0.14523 (13)	0.62214 (11)	0.65281 (7)	0.0358 (3)	
C20	0.25545 (13)	0.55958 (10)	0.62526 (7)	0.0345 (3)	
C21	0.39212 (13)	0.60103 (10)	0.66650 (7)	0.0345 (3)	
C22	0.49792 (14)	0.46536 (11)	0.60637 (7)	0.0382 (3)	
C23	0.36232 (14)	0.42444 (11)	0.56559 (7)	0.0378 (3)	
C24	0.35458 (17)	0.32749 (12)	0.51582 (8)	0.0483 (3)	
H24	0.2661	0.2993	0.4894	0.058*	
C25	0.47617 (18)	0.27492 (13)	0.50633 (9)	0.0547 (4)	
H25	0.4701	0.2115	0.4730	0.066*	
C26	0.61001 (17)	0.31559 (13)	0.54628 (9)	0.0547 (4)	
H26	0.6921	0.2787	0.5394	0.066*	
C27	0.62156 (15)	0.40861 (12)	0.59517 (9)	0.0481 (3)	
H27	0.7112	0.4348	0.6213	0.058*	

### Atomic displacement parameters (Å^2^)

**Table:**

	*U*^11^	*U*^22^	*U*^33^	*U*^12^	*U*^13^	*U*^23^
N1	0.0374 (6)	0.0388 (6)	0.0444 (6)	0.0031 (5)	0.0144 (5)	0.0002 (5)
N2	0.0425 (6)	0.0388 (6)	0.0375 (6)	0.0012 (5)	0.0099 (5)	−0.0035 (5)
C1	0.0864 (13)	0.0474 (9)	0.0773 (12)	−0.0137 (9)	0.0241 (10)	0.0124 (9)
C2	0.0785 (12)	0.0502 (9)	0.0802 (13)	0.0038 (9)	0.0069 (10)	0.0227 (9)
C3	0.0501 (8)	0.0495 (8)	0.0577 (9)	0.0020 (7)	0.0027 (7)	0.0076 (7)
C4	0.0425 (7)	0.0346 (6)	0.0354 (7)	0.0000 (5)	0.0093 (5)	−0.0031 (5)
C5	0.0438 (8)	0.0458 (8)	0.0601 (9)	0.0000 (6)	0.0143 (7)	0.0061 (7)
C6	0.0559 (9)	0.0569 (10)	0.0786 (12)	−0.0120 (8)	0.0201 (8)	0.0063 (9)
C7	0.0323 (6)	0.0350 (6)	0.0380 (7)	0.0017 (5)	0.0088 (5)	−0.0035 (5)
C8	0.0323 (6)	0.0451 (7)	0.0379 (7)	−0.0007 (5)	0.0097 (5)	0.0017 (6)
C9	0.0470 (8)	0.0578 (9)	0.0422 (8)	0.0025 (7)	0.0076 (6)	−0.0065 (7)
C10	0.0554 (9)	0.0887 (13)	0.0408 (9)	−0.0076 (9)	0.0087 (7)	−0.0100 (8)
C11	0.0606 (10)	0.1086 (15)	0.0401 (9)	−0.0102 (10)	0.0062 (7)	0.0181 (10)
C12	0.0644 (11)	0.0752 (12)	0.0589 (11)	0.0029 (9)	0.0113 (8)	0.0293 (9)
C13	0.0507 (8)	0.0505 (8)	0.0513 (9)	0.0006 (7)	0.0146 (7)	0.0102 (7)
C14	0.0349 (6)	0.0364 (6)	0.0381 (7)	0.0029 (5)	0.0095 (5)	0.0006 (5)
C15	0.0424 (7)	0.0459 (7)	0.0473 (8)	0.0032 (6)	0.0116 (6)	−0.0110 (6)
C16	0.0426 (7)	0.0523 (8)	0.0544 (9)	0.0082 (6)	0.0183 (6)	−0.0072 (7)
C17	0.0327 (7)	0.0566 (8)	0.0568 (9)	0.0031 (6)	0.0108 (6)	−0.0035 (7)
C18	0.0368 (7)	0.0460 (7)	0.0460 (8)	0.0004 (6)	0.0068 (6)	−0.0057 (6)
C19	0.0350 (6)	0.0361 (6)	0.0365 (7)	0.0025 (5)	0.0088 (5)	0.0001 (5)
C20	0.0363 (6)	0.0336 (6)	0.0341 (7)	0.0010 (5)	0.0093 (5)	0.0012 (5)
C21	0.0368 (6)	0.0324 (6)	0.0357 (6)	0.0013 (5)	0.0114 (5)	0.0015 (5)
C22	0.0446 (7)	0.0339 (6)	0.0398 (7)	0.0043 (5)	0.0174 (6)	0.0052 (5)
C23	0.0465 (7)	0.0356 (6)	0.0335 (7)	0.0049 (5)	0.0137 (5)	0.0031 (5)
C24	0.0601 (9)	0.0433 (7)	0.0426 (8)	0.0030 (6)	0.0143 (6)	−0.0052 (6)
C25	0.0788 (11)	0.0408 (8)	0.0509 (9)	0.0103 (7)	0.0278 (8)	−0.0044 (7)
C26	0.0614 (9)	0.0458 (8)	0.0657 (10)	0.0147 (7)	0.0324 (8)	0.0033 (7)
C27	0.0457 (8)	0.0454 (8)	0.0582 (9)	0.0069 (6)	0.0225 (7)	0.0040 (7)

### Geometric parameters (Å, º)

**Table:**

N1—C21	1.3038 (16)	C11—C12	1.371 (3)
N1—C22	1.3791 (17)	C12—H12	0.9300
N2—C20	1.3099 (16)	C12—C13	1.386 (2)
N2—C23	1.3744 (17)	C13—H13	0.9300
C1—H1	0.9300	C14—C15	1.3878 (18)
C1—C2	1.368 (3)	C14—C19	1.3984 (18)
C1—C6	1.371 (2)	C15—H15	0.9300
C2—H2	0.9300	C15—C16	1.384 (2)
C2—C3	1.383 (2)	C16—H16	0.9300
C3—H3	0.9300	C16—C17	1.384 (2)
C3—C4	1.3790 (19)	C17—H17	0.9300
C4—C5	1.3925 (19)	C17—C18	1.3826 (19)
C4—C7	1.5375 (18)	C18—H18	0.9300
C5—H5	0.9300	C18—C19	1.3909 (19)
C5—C6	1.376 (2)	C19—C20	1.4583 (17)
C6—H6	0.9300	C20—C21	1.4303 (18)
C7—C8	1.5276 (19)	C22—C23	1.4176 (19)
C7—C14	1.5408 (18)	C22—C27	1.4116 (18)
C7—C21	1.5353 (17)	C23—C24	1.4097 (18)
C8—C9	1.390 (2)	C24—H24	0.9300
C8—C13	1.384 (2)	C24—C25	1.362 (2)
C9—H9	0.9300	C25—H25	0.9300
C9—C10	1.382 (2)	C25—C26	1.399 (2)
C10—H10	0.9300	C26—H26	0.9300
C10—C11	1.371 (3)	C26—C27	1.362 (2)
C11—H11	0.9300	C27—H27	0.9300
			
C21—N1—C22	114.68 (11)	C12—C13—H13	119.8
C20—N2—C23	114.37 (11)	C15—C14—C7	128.73 (12)
C2—C1—H1	120.2	C15—C14—C19	119.19 (12)
C2—C1—C6	119.53 (15)	C19—C14—C7	112.03 (10)
C6—C1—H1	120.2	C14—C15—H15	120.4
C1—C2—H2	119.8	C16—C15—C14	119.16 (13)
C1—C2—C3	120.42 (16)	C16—C15—H15	120.4
C3—C2—H2	119.8	C15—C16—H16	119.3
C2—C3—H3	119.6	C15—C16—C17	121.38 (12)
C4—C3—C2	120.81 (15)	C17—C16—H16	119.3
C4—C3—H3	119.6	C16—C17—H17	119.9
C3—C4—C5	118.09 (13)	C18—C17—C16	120.25 (13)
C3—C4—C7	122.92 (12)	C18—C17—H17	119.9
C5—C4—C7	118.97 (11)	C17—C18—H18	120.7
C4—C5—H5	119.7	C17—C18—C19	118.52 (13)
C6—C5—C4	120.69 (14)	C19—C18—H18	120.7
C6—C5—H5	119.7	C14—C19—C20	108.37 (11)
C1—C6—C5	120.45 (16)	C18—C19—C14	121.48 (11)
C1—C6—H6	119.8	C18—C19—C20	130.03 (12)
C5—C6—H6	119.8	N2—C20—C19	127.41 (11)
C4—C7—C14	113.08 (10)	N2—C20—C21	123.76 (11)
C8—C7—C4	110.86 (10)	C21—C20—C19	108.72 (11)
C8—C7—C14	110.09 (10)	N1—C21—C7	126.20 (11)
C8—C7—C21	114.09 (10)	N1—C21—C20	123.29 (12)
C21—C7—C4	108.26 (10)	C20—C21—C7	110.51 (10)
C21—C7—C14	100.11 (9)	N1—C22—C23	121.89 (11)
C9—C8—C7	118.92 (12)	N1—C22—C27	119.16 (12)
C13—C8—C7	122.69 (12)	C27—C22—C23	118.94 (13)
C13—C8—C9	118.32 (13)	N2—C23—C22	121.97 (12)
C8—C9—H9	119.5	N2—C23—C24	118.80 (12)
C10—C9—C8	121.01 (15)	C24—C23—C22	119.20 (12)
C10—C9—H9	119.5	C23—C24—H24	119.9
C9—C10—H10	120.1	C25—C24—C23	120.28 (14)
C11—C10—C9	119.83 (16)	C25—C24—H24	119.9
C11—C10—H10	120.1	C24—C25—H25	119.7
C10—C11—H11	120.0	C24—C25—C26	120.55 (14)
C10—C11—C12	120.03 (16)	C26—C25—H25	119.7
C12—C11—H11	120.0	C25—C26—H26	119.6
C11—C12—H12	119.8	C27—C26—C25	120.79 (14)
C11—C12—C13	120.41 (16)	C27—C26—H26	119.6
C13—C12—H12	119.8	C22—C27—H27	119.9
C8—C13—C12	120.35 (15)	C26—C27—C22	120.24 (14)
C8—C13—H13	119.8	C26—C27—H27	119.9
			
C6—C1—C2—C3	−0.9 (3)	C17—C18—C19—C14	−1.3 (2)
C1—C2—C3—C4	0.0 (3)	C17—C18—C19—C20	174.12 (13)
C2—C3—C4—C5	0.7 (2)	C15—C14—C19—C18	1.67 (19)
C2—C3—C4—C7	−178.05 (15)	C7—C14—C19—C18	179.30 (11)
C3—C4—C5—C6	−0.5 (2)	C15—C14—C19—C20	−174.64 (11)
C7—C4—C5—C6	178.31 (14)	C7—C14—C19—C20	2.99 (14)
C2—C1—C6—C5	1.1 (3)	C23—N2—C20—C21	1.74 (17)
C4—C5—C6—C1	−0.4 (3)	C23—N2—C20—C19	−173.95 (11)
C3—C4—C7—C8	−131.10 (13)	C18—C19—C20—N2	0.7 (2)
C5—C4—C7—C8	50.19 (15)	C14—C19—C20—N2	176.62 (12)
C3—C4—C7—C21	103.05 (14)	C18—C19—C20—C21	−175.49 (13)
C5—C4—C7—C21	−75.66 (14)	C14—C19—C20—C21	0.40 (14)
C3—C4—C7—C14	−6.91 (17)	C22—N1—C21—C20	−1.39 (17)
C5—C4—C7—C14	174.37 (11)	C22—N1—C21—C7	178.39 (11)
C21—C7—C8—C13	−13.19 (16)	N2—C20—C21—N1	−0.19 (19)
C4—C7—C8—C13	−135.71 (13)	C19—C20—C21—N1	176.19 (11)
C14—C7—C8—C13	98.41 (14)	N2—C20—C21—C7	180.00 (11)
C21—C7—C8—C9	169.96 (11)	C19—C20—C21—C7	−3.62 (13)
C4—C7—C8—C9	47.44 (15)	C8—C7—C21—N1	−57.34 (16)
C14—C7—C8—C9	−78.44 (14)	C4—C7—C21—N1	66.59 (15)
C13—C8—C9—C10	−1.1 (2)	C14—C7—C21—N1	−174.85 (12)
C7—C8—C9—C10	175.84 (12)	C8—C7—C21—C20	122.46 (11)
C8—C9—C10—C11	1.4 (2)	C4—C7—C21—C20	−113.61 (11)
C9—C10—C11—C12	−0.1 (3)	C14—C7—C21—C20	4.95 (12)
C10—C11—C12—C13	−1.5 (3)	C21—N1—C22—C27	−177.33 (11)
C9—C8—C13—C12	−0.4 (2)	C21—N1—C22—C23	1.35 (17)
C7—C8—C13—C12	−177.30 (13)	C20—N2—C23—C24	176.38 (11)
C11—C12—C13—C8	1.8 (2)	C20—N2—C23—C22	−1.72 (17)
C8—C7—C14—C15	52.10 (17)	N1—C22—C23—N2	0.21 (19)
C21—C7—C14—C15	172.54 (13)	C27—C22—C23—N2	178.89 (11)
C4—C7—C14—C15	−72.51 (17)	N1—C22—C23—C24	−177.89 (11)
C8—C7—C14—C19	−125.24 (12)	C27—C22—C23—C24	0.79 (18)
C21—C7—C14—C19	−4.80 (13)	N2—C23—C24—C25	−179.05 (13)
C4—C7—C14—C19	110.15 (12)	C22—C23—C24—C25	−0.9 (2)
C19—C14—C15—C16	−0.8 (2)	C23—C24—C25—C26	0.6 (2)
C7—C14—C15—C16	−177.99 (13)	C24—C25—C26—C27	−0.2 (2)
C14—C15—C16—C17	−0.4 (2)	C25—C26—C27—C22	0.1 (2)
C15—C16—C17—C18	0.7 (2)	N1—C22—C27—C26	178.29 (12)
C16—C17—C18—C19	0.1 (2)	C23—C22—C27—C26	−0.4 (2)

### Hydrogen-bond geometry (Å, º)

*Cg*2 and *Cg*3 are the centroids of the N1/N2/C20–C23 and C1–C6 rings, respectively.

**Table:**

*D*—H···*A*	*D*—H	H···*A*	*D*···*A*	*D*—H···*A*
C15—H15···*Cg*2^i^	0.93	2.92	3.728 (2)	145
C25—H25···*Cg*3^ii^	0.93	2.96	3.830 (3)	156

Symmetry codes: (i) −*x*+1/2, *y*+1/2, −*z*+3/2; (ii) −*x*+1, −*y*+1, −*z*+1.
